# Response Surface Methodology (RSM) for Optimizing Protein Extraction from Housefly (*Musca domestica*) Larvae Fed with Toad and Its Structural Characterization

**DOI:** 10.3390/molecules29112595

**Published:** 2024-05-31

**Authors:** Jingnan Miao, Chenglu Yu, Xianhe Cheng, Junqiang Qiu, Shumin Liu

**Affiliations:** 1 Graduate School, Institute of Traditional Chinese Medicine, Heilongjiang University of Chinese Medicine, Harbin 150040, China; mjn_788@126.com (J.M.); yuchenglu100@163.com (C.Y.); chengxianhe_up_up@163.com (X.C.); 2 Key Laboratory of Tropical Translational Medicine of Ministry of Education, International Joint Research Center of Human-Machine Intelligent Collaborative for Tumor Precision Diagnosis and Treatment of Hainan Province, School of Pharmacy, Hainan Medical University, Haikou 570100, China

**Keywords:** *Musca domestica* L., proteins, optimization, characterization

## Abstract

With the global population on the rise, an escalating interest exists in environmentally sustainable and friendly protein sources. Insects have emerged as multifaceted resources, viewed not only as potential food items, but also as sources of traditional medicines and proteins. This study utilized response surface methodology (RSM) to ascertain the optimal extraction conditions for proteins from *Musca domestica* used in toad feeding, denoted as MDPs-T. The yield of MDPs-T was elevated to 18.3% ± 0.2% under these optimized conditions. Subsequently, the particle size, ζ-potentials, and structures of MDPs-T were analyzed and compared with the proteins derived from *Musca domestica* fed on a normal diet (MDPs-ND). This comparative analysis utilized a range of advanced techniques, involving UV spectroscopy, Fourier transform infrared spectroscopy (FT-IR), X-ray diffraction (XRD), high-performance gel permeation chromatography (HPGPC), and scanning electron microscopy (SEM). The outcomes have revealed a marginal disparity in the physical and chemical properties between MDPs-T and MDPs-ND. Derosination led to a reduction in the particle size of the MDPs by 10.98% to 62.81%. MDPs-T exhibited a higher proportion of low-molecular-weight components relative to MDPs-ND. Additionally, in a comparative analysis of amino acids, MDPs-T displayed a greater abundance of essential and total amino acids relative to MDPs-ND. Consequently, MDPs-T holds potential as a valuable food supplement for human consumption or as a nutrient-rich feed supplement for animals.

## 1. Introduction

In numerous countries across Asia, Africa, and the Americas, various insects and their byproducts are commonly employed in traditional medicine or as functional foods. Examples include China, India, South Korea, Congo, Zimbabwe, Brazil, and Mexico. For instance, thousands of traditional Chinese medicine (TCM) remedies feature edible insects and insect-derived raw materials. In Western nations, the primary focus of insect farming is largely geared towards producing animal feed [1]. The rising interest in using insects for animal nutrition is notable, due to EU regulations [2].

In addition, the world’s human population continues to grow, and is projected to reach 9.7 billion by 2050. Hence, the worldwide demand for food is poised to noticeably rise. This underscores the urgent requirement for novel food sources to augment the existing array of traditional proteins. Insect proteins are widely recognized as potentially excellent alternatives to traditional proteins, due to their high feed conversion ratio, protein content exceeding 35%, minimal resource consumption, and low greenhouse gas emissions. Additionally, insects are rich in various essential nutrients, such as lipids, fatty acids, minerals, and vitamins. As previously mentioned, the notable interest in insect proteins is driven by the environmental pressures associated with the ever-expanding human population [3].

Among the array of edible insects, the larvae of the housefly (*Musca domestica*, *M. domestica*) emerge as a particularly promising functional food resource. Their historical significance in TCM dates back to the 14th century, where they were utilized for a spectrum of ailments, such as coma, decubital necrosis, ecthyma, gastric cancer, lip scald, malnutritional stagnation, and osteomyelitis. Since 1917, these larvae have been intentionally bred for livestock feed. Interestingly, their ability to flourish even in unhygienic environments is attributable to a diverse array of active components, including proteins, peptides, polysaccharides, polyunsaturated fats, lysozyme, fiber, minerals, vitamins, and agglutinin [4,5]. Within this realm of possibilities, the functional proteins and peptides sourced from *M. domestica* have garnered substantial interest for their potential applications across pharmaceuticals, foods, and cosmetics. An in-depth examination of their composition has unveiled that M. domestica larvae boast a noteworthy protein content exceeding 45% in dry weight, which are notably abundant in vital amino acids, such as lysine and methionine. These *M. domestica* proteins (MDPs) showcase a myriad of bioactivities, ranging from their antimicrobial prowess [6] and anti-tumor [7] properties to their antioxidant capacities [8] and ability to combat atherosclerosis [9]. They also exhibit anti-inflammatory attributes [10], antiviral potential [11], efficacy against diarrhea [12], the modulation of beneficial intestinal microflora [13], and hepatoprotective benefits [14]. Nonetheless, despite being the primary active constituents in *M. domestica*, MDPs have been infrequently reported on in the past decade. An escalating number of scholars have recently documented alkali extraction and acid precipitation methods for isolating proteins from natural sources, such as insects, mushrooms, and plants [15]. Multiple investigations have highlighted that these alkali extraction and acid precipitation techniques could serve as cost-effective and practical means of protein extraction in industrial settings, owing to their high recovery rates and low costs [16]. This technique is typically employed to enhance the release and dissolution of proteins during the extraction process, primarily by disrupting the dense structure of natural products to increase the protein yield [17,18,19].

As it stands, there has been no exploration into the extraction, separation, purification, and structural characterization of MDPs derived from *M. domestica* larvae fed with toad (MDPs-T). While some studies have touched upon the extraction process, chemical composition, and pharmacological activities of MDPs extracted using distilled water, acid, and deep eutectic solvents [20,21], there is a noticeable absence of research on the alkali water extraction of MDPs. Therefore, it represents a meaningful undertaking for us to concentrate on this investigation in order to unearth significant discoveries concerning MDPs-T using alkaline water [22].

In this investigation, we attempted to utilize the response surface methodology (RSM) based on a Box–Behnken design (BBD) to determine the ideal extraction parameters (including pH, MTL ratio, time, and temperature) for MDPs-T. The consensus among experts is that the BBD offers superior fitting for the quadratic model relative to other experimental designs, boasting higher efficiency and lower costs. Following this optimization, the crude MDPs-T underwent a purification process using the acid precipitation method. To gain insights into the structural characteristics of the MDPs, a range of sophisticated techniques were employed, including UV spectroscopy, Fourier transform infrared (FT-IR) spectroscopy, high-performance gel permeation chromatography (HPGPC), X-ray diffraction (XRD), and scanning electron microscopy (SEM). Furthermore, a detailed comparative analysis was undertaken between MDPs-T and flies fed a regular diet (MDPs-ND). This pioneering study represents the first instance of reporting the optimal extraction conditions for MDPs from *M. domestica* larvae fed with toad, thereby making a substantial advancement in this area of research.

## 2. Results and Discussion

### 2.1. Influences of Different Extraction Conditions on the Extraction Rate of MDPs

The alkaline extraction and acid precipitation methods stand out as prominent techniques for extracting proteins from natural sources, providing a plethora of advantages, including cost-effectiveness, simplicity of operation, and the absence of specialized equipment. These attributes render them highly appropriate for large-scale industrial applications.

**Figure 1:**
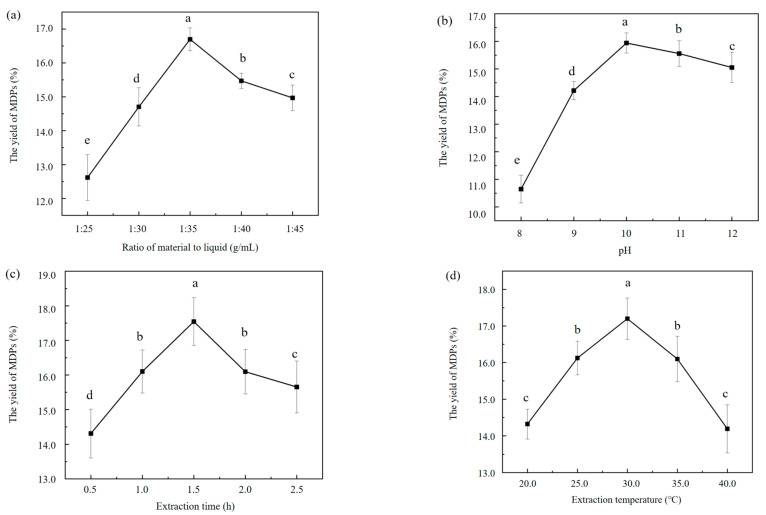
Effect of different extraction parameters on the extraction rate of MDPs-T. (**a**) Ratio of material to liquid, g/mL; (**b**) pH; (**c**) Extraction time, h; (**d**) Extraction temperature, °C. Each value represents the mean ± SD of triplicates. Different letters represent significant differences between two groups.

This study figured out the notable influences of varying MTL ratios on the extraction efficiency of MDPs (Figure 1a). The experimental parameters were rigorously controlled, as follows: the pH was maintained at 10.0, the temperature was maintained at 30.0 °C, and the duration was 2.0 h. Evidently, the MTL ratio wielded a significant influence on the extraction efficiency of the MDPs. The outcomes emphasized an escalating extraction efficiency of MDPs with an increasing MTL ratio. Specifically, at a ratio of 1:35.0 g/mL, the extraction efficiency surged notably to 16.8% ± 1.3%. However, upon further amplification of the MTL ratio, a marginal decline in the extraction efficiency of the MDPs was discerned. Therefore, the optimal MTL ratio of 1:35.0 g/mL was utilized for subsequent investigations. Remarkably, these outcomes exhibited a striking resemblance to the yield range of soy protein obtained elsewhere (range, 14.5–21.5%) [23].

In a comprehensive evaluation of diverse pH conditions on the extraction efficacy of MDPs, extractions were undertaken across a spectrum of pH values ranging from 8 to 12. The experimental setup maintained the other parameters at an MTL ratio of 1:35.0 g/mL, a duration of 2.0 h, and an extraction temperature of 30.0 °C. The outcomes illuminated a notable elevation (*p* < 0.05) in the extraction efficiency of the MDPs as the pH was escalated from 8.0 to 10.0. This upward trend culminated in the peak extraction efficiency of MDPs at a pH of 10.0, standing at 15.9% ± 0.9%. It is noteworthy that, as the pH further ascended from 10.0 to 12.0, there was a discernible yet slight decrease (*p* < 0.05) in the extraction efficiency of the MDPs (Figure 1b). Increasing the pH led to the improved solubility of MDPs. The pH was too high, which led to protein denaturation, and, thus, a decrease in the extraction of MDPs. Alkaline extraction has been proven to increase the protein yield of natural products [24].

To thoroughly figure out the influences of various extraction durations on the extraction efficiency of MDPs, we attempted to set the extraction time to 0.5, 1.0, 1.5, 2.0, and 2.5 h. All of the other parameters were precisely maintained as follows: a pH of 10.0, an MTL ratio of 1:35.0 g/mL, and an extraction temperature of 30.0 ° C. The graphical representation in Figure 1c illuminates the influences of the different extraction durations on the extraction efficiency of MDPs. Evidently, the extraction rate exhibited a consistent upward trend with extending extraction duration. At an extraction time of 1.5 h, the extraction efficiency peaked significantly at 17.5% ± 1.3%. However, upon further extension of the extraction duration beyond this optimal point, a notable decline in the extraction efficiency of MDPs was identified. A similar trend for extraction yield and extraction duration was observed by Ortega et al. [25].

It has been reported that natural products are prone to the denaturation of proteins during heat-stabilized processing, which results in a reduction in soluble proteins. Ultimately, the influences of extraction temperature on the extraction efficiency of MDPs are elucidated in Figure 1d, where we attempted to precisely set temperatures at 20.0, 25.0, 30.0, 35.0, and 40.0 °C, respectively. The remaining parameters were rigorously maintained as follows: a pH of 10.0, an MTL ratio of 1:35.0 g/mL, and an extraction duration of 1.5 h. This comprehensive examination underscores the notable function of temperature in the extraction process of MDPs. The outcomes unveiled a distinct trend in the extraction efficiency with varying temperatures. Notably, the extraction rate exhibited a substantial ascent from 20.0 to 30.0 °C, followed by a gradual decline with further temperature increments. It is noteworthy that, at the optimal temperature of 30.0 °C, the extraction efficiency of the MDPs soared to its zenith, at 17.3% ± 1.6%. As the temperature increases, the protein solubility increases, resulting in the improvement of protein extraction efficiency. In contrast, excessively high temperatures may cause the protein’s molecular structure to collapse and then precipitate, resulting in a decrease in extraction efficiency [26]. These outcomes highlight the importance of precise temperature control in optimizing the extraction of MDPs.

### 2.2. Optimizing Extraction Parameters of MDPs

#### 2.2.1. Model Fitting

The response value, specifically the extraction rate of MDPs under diverse experimental conditions and their interactions, is outlined in Table 1. The Box–Behnken design, consisting of four factors at three levels each, was employed to figure out the influences of various extraction parameters (pH, MTL ratio, time, and temperature) on the extraction rate of MDPs. As part of the design, we attempted to conduct five replicates, particularly at the central point. Additionally, utilizing five center-point runs, we attempted to verify the stability and intrinsic variability of the extraction experiment.

#### 2.2.2. Fitting and Assessing the Adequacy of the Model

The experimental data were processed on the basis of multivariate regression analysis. A second-order polynomial model was utilized for the regression analysis, formulating the relationship of the independent response variable (*Y*) with the dependent response variables (*X*), as follows:

*Y* = 18.5412 + 0.4506*X*_1_ + 0.7107*X*_2_ − 0.3287*X*_3_ + 0.3791*X*_4_ − 0.0228*X*_1_*X*_2_ − 0.1019*X*_1_*X*_3_ − 0.0879*X*_1_*X*_4_ − 0.0291*X*_2_*X*_3_ − 0.5971*X*_2_*X*_4_ − 0.6952*X*_3_*X*_4_ − 1.6017*X*_1_^2^ − 1.1589*X*_2_^2^ − 0.7639*X*_3_^2^ − 1.5374*X*_4_^2^

where *X*_1_ is the MTL ratio (g/mL), *X*_2_ is the pH, *X*_3_ is the time (hour), and *X*_4_ is the temperature (°C).

Upon evaluating the ANOVA outcomes delineated in Table 2, the model exhibited an F value of 14.93 (*p* < 0.0001), denoting an exceedingly significant response to the extraction rate of MDPs. Furthermore, we attempted to calculate the P value for the absence of fit as 0.6304 (*p* > 0.05), suggesting a well-fitted model. This confirmation of the model’s reliability was pivotal.

Delving deeper, the coefficient of determination (R^2^) was computed to be 0.9372, accompanied by an adjusted determination coefficient (R^2^_adj_) of 0.8745. These figures portray a robust concordance between the model and both the theoretical and practical data concerning the extraction rate of MDPs. The absence of fit result (*p* > 0.05) is noteworthy, indicating the model’s adeptness in representing the practical data without substantial discrepancies, thereby reducing the likelihood of noise interference. Therefore, the model is deemed fitting for the results. Additionally, the coefficient of variation (C.V.) was notably minimal at 2.82%, signifying a commendable level of reproducibility and reliability in the experimental values. Upon closer examination of Table 2, it is evident that all linear coefficients (X_1_, X_2_, X_3_, and X_4_), quadratic term coefficients (X_1_^2^, X_2_^2^, X_3_^2^, and X_4_^2^), and the cross-product coefficients (X_2_X_4_ and X_3_X_4_) exhibited statistical significance (*p* < 0.05). This comprehensive analysis underscores the intricate relationship between the extraction parameters and the extraction rate of MDPs. In addition, the F value confirmed that the order of factors affecting the extraction rate of the MDPs was arranged as follows: pH > extraction temperature > MTL ratio > time. Moreover, the order of the interaction effects was arranged as follows: X_3_X_4_ > X_2_X_4_ > X_1_X_3_ > X_1_X_4_ > X_2_X_3_ > X_1_X_2_.

#### 2.2.3. Response Surface Analysis

**Figure 2:**
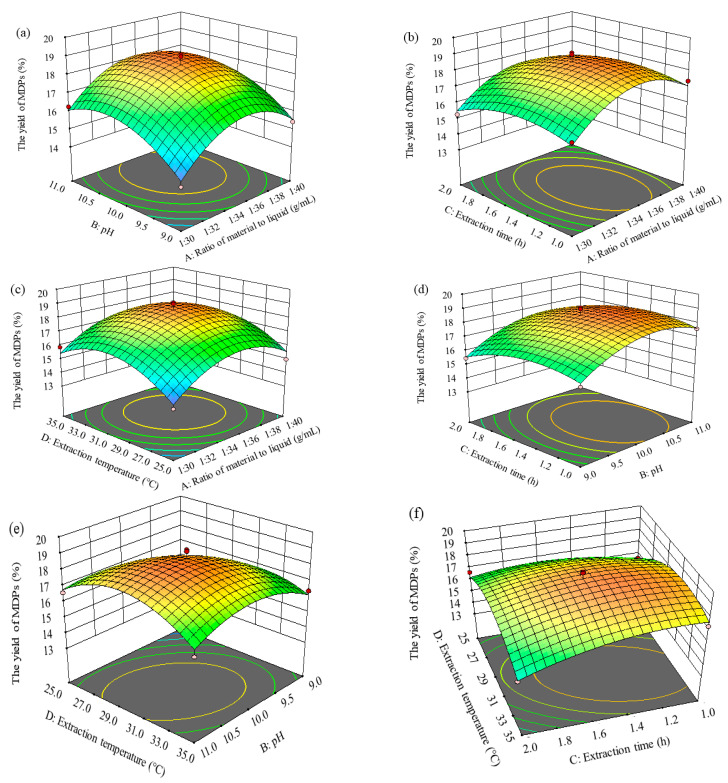
Response surface plots showing the interactions of different selected factors. (**a**) Effect of ratio of material to liquid and pH on the extraction rate of MDPs-T; (**b**) Effect of ratio of material to liquid and extraction time on the extraction rate of MDPs-T; (**c**) Effect of ratio of material to liquid and extraction temperature on the extraction rate of MDPs-T; (**d**) Effect of pH and extraction time on the extraction rate of MDPs; (**e**) Effect of pH and extraction temperature on the extraction rate of MDPs-T; (**f**) Effect of extraction time and extraction temperature on the extraction rate of MDPs-T.

**Figure 3:**
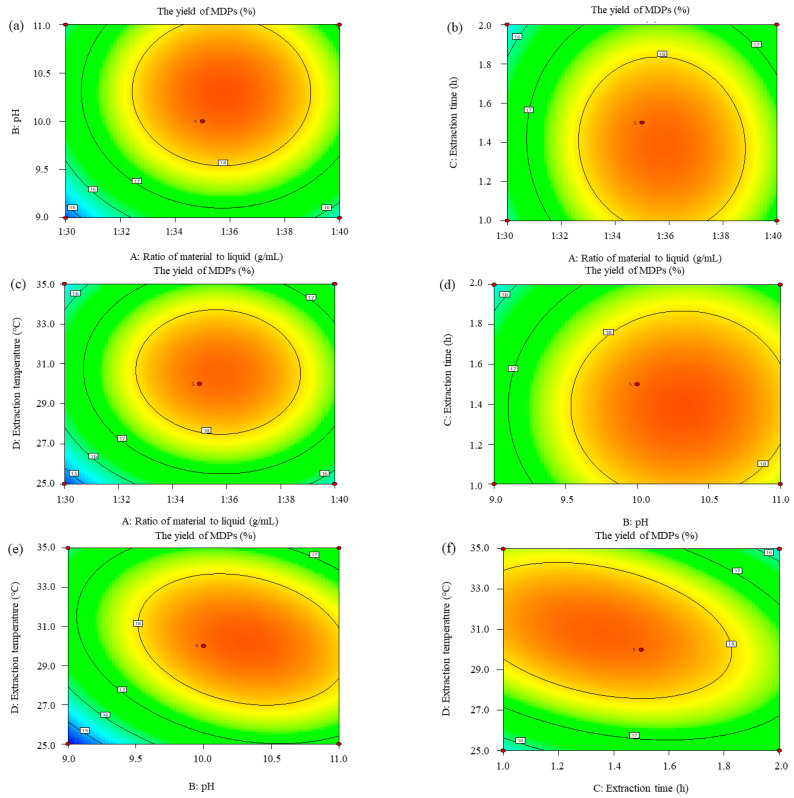
Contour plots displaying the effects of different extraction parameters on the extraction rate of MDPs-T. (**a**) Effect of ratio of material to liquid and pH on the extraction rate of MDPs-T; (**b**) Effect of ratio of material to liquid and extraction time on the extraction rate of MDPs-T; (**c**) Effect of ratio of material to liquid and extraction temperature on the extraction rate of MDPs-T; (**d**) Effect of pH and extraction time on the extraction rate of MDPs-T; (**e**) Effect of pH and extraction temperature on the extraction rate of MDPs; (**f**) Effect of extraction time and extraction temperature on the extraction rate of MDPs-T.

To attain notable insights into the influences of independent variables on the response, we concentrated on the analysis utilizing the model’s 3D response surface plots. These plots were precisely examined to discern the optimal values for achieving the pinnacle extraction rate of MDPs. The comprehensive evaluation employed both the 3D response surface plots (Figure 2) and the contour plots (Figure 3) to precisely figure out the intricate correlation among the various variables. The visual depiction of these interrelations was derived from the pronounced steepness observed within the 3D plot, serving as a visual cue for the depth of interactions between the two experimental variables.

Figure 2a–c and Figure 3a–c elucidate the multifaceted influences of pH and the MTL ratio, time and the MTL ratio, and temperature and the MTL ratio on the extraction rate of MDPs. The discernments gleaned from these depictions underscore that the impact of the MTL ratio on the extraction rate is larger in significance relative to the influences of pH, temperature, and time on the intricate curved surface. Specifically, the influence of the MTL ratio on the extraction rate stands out as being particularly notable. An exponential rise in the extraction rate is evident as the MTL ratio ascends from 1:30.0 to 1:36.0. However, as this ratio further escalates from 1:36.0 to 1:40.0, concurrently with the pH shifting from 10.0 to 11.0, the temperature rising from 31.0 to 35.0 °C, and the time span stretching from 1.4 to 2.0 h, a significant plummet in the extraction rate of MDPs is observed, indicating intricate and nonlinear relationships among these variables.

The impacts of pH and time, as well as extraction temperature, on the extraction rate of MDPs are depicted in Figure 2d and Figure 3d and Figure 2e and Figure 3e, respectively. These findings reveal that the influences of pH on the extraction rate of MDPs escalated more swiftly than those of time and temperature, evidenced by the notably steeper curved surface for pH. The extraction rate exhibited a rapid increase with the elevation of pH from 9.0 to 10.0, whereas it rose gradually with the increase in extraction time from 1.0 to 1.6 h and extraction temperature from 25.0 to 29.0 °C.

Moving on to the investigation presented in Figure 2f and Figure 3f, we examined the impacts of extraction time and extraction temperature on the extraction rate. Notably, the extraction rate of MDPs exhibited a moderate increase with the escalation of these interaction factors. The assessment of the significance of the variables’ interaction was on the basis of the steepness of the response surface, a measure consistent with the ANOVA outcomes.

#### 2.2.4. Model Verification

The culmination of optimal conditions to maximize the extraction rate of MDPs was achieved through model analysis as follows: a precisely tuned MTL ratio of 1:36.0 g/mL, a pH maintained at 10.3, an extraction temperature carefully set at 30.5 °C, and an extraction duration of 1.3 h. This calculated point was rigorously tested under real-world optimal settings, confirming an MTL ratio of 1:36.0 g/mL, a pH adjusted to 10, an extraction temperature precisely controlled at 31.0 °C, and an extraction time of 1.3 h. Under these precisely fine-tuned conditions, the extraction rate of the MDPs was found to be 18.3% ± 0.1%. It is of particular significance to note that, upon comparison with the anticipated value of 18.5%, the statistical assay revealed no significant deviation (*p* > 0.05). This robustly supports the model’s validity and its ability to accurately predict the optimal extraction conditions for maximizing the yield of MDPs.

### 2.3. Structural Characteristics

#### 2.3.1. Particle Sizing and Zeta Potential

As illustrated in Table 3, the particle sizes of MDPs-T and MDPs-ND were measured at 246.4 ± 5.6 and 363.2 ± 16.6 nm, respectively. Conversely, the particle sizes of the defatted MDPs-T and the defatted MDPs-ND were 219.3 ± 31.1 and 135.7 ± 3.3 nm, respectively. These findings reveal that the defatting process led to a reduction in the particle size of MDPs, ranging from 10.98% to 62.81%. This reduction indicates a notable alteration in the physical characteristics of MDPs due to defatting, resulting in a decreased particle size. The decrease in protein particle size can significantly influence its functional and conformational properties.

Zeta potential, a critical parameter for evaluating the dispersion characteristics of a polymer solution or colloid, plays a notable function in assessing stability. A higher absolute value of zeta potential indicates smaller protein molecules scattered within the solution and a more stable system. When comparing defatted MDPs-ND to MDPs-ND, a remarkable 24.32% increase in the absolute surface charge was identified. However, the absolute surface charges of MDPs-T and MDPs-ND remained relatively consistent at −39.0 ± 1.5 and −37.0 ± 1.7, respectively. This indicates that, while defatting notably influenced the zeta potential of MDPs-ND, the original MDPs-T maintained a consistent zeta potential with MDPs-ND [27].

#### 2.3.2. Ultraviolet–Visible (UV–Vis) Spectra and Intrinsic Fluorescence Spectra

**Figure 4:**
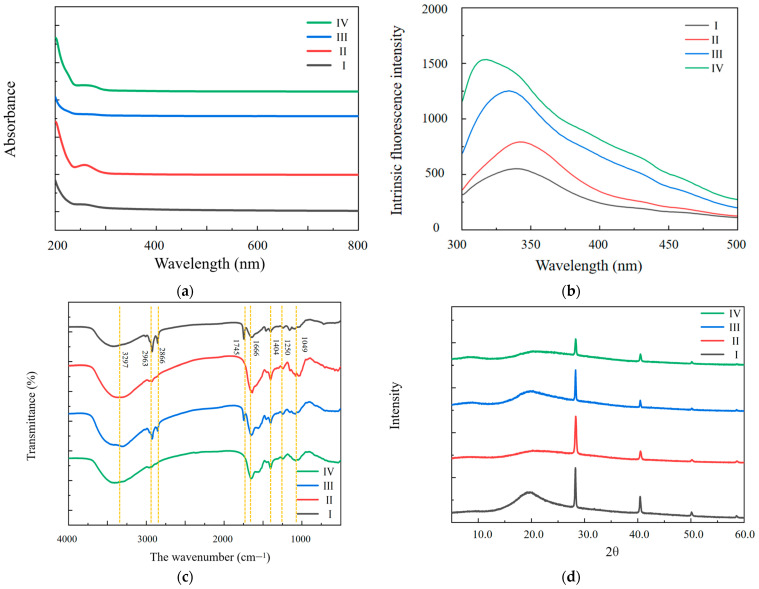
The characterization of MDPs. (**a**) The UV spectrum of MDPs; (**b**) Fluorescence spectra of MDPs; (**c**) The FTIR spectra of MDPs; (**d**) X-ray diffraction pattern, I: MDPs-ND, II: Defatted MDPs-ND, III: MDPs-T, IV: Defatted MDPs-T.

UV–Vis absorption and intrinsic fluorescence are exquisitely attuned to alterations in proteins or peptides, primarily driven by the presence of aromatic amino acids, such as phenylalanine, tyrosine, and tryptophan. As illustrated in Figure 4a, the process of defatting induced a significant enhancement in UV–Vis absorption intensity, which was particularly evident within the 240 to 290 nm range for both defatted MDPs-T and defatted MDPs-ND. This augmentation resulted in visibly larger absorption peaks for both defatted variants. The maximum absorption wavelength at 270 nm can be attributed to the vibrational characteristics of amino acid residues present in MDPs, a phenomenon also noted in the soy protein study conducted by Banerjee et al. [15].

Fluorescence spectroscopy of MDPs serves as a powerful tool to discern the nuanced conformational and structural changes within its tertiary framework, particularly concerning the presence of chromophores, such as phenylalanine, tyrosine, and tryptophan. As delineated in Figure 4b, both defatted MDPs-T and defatted MDPs-ND exhibit a discernibly heightened fluorescence intensity across the spectral range of 300 to 375 nm. Notably, an evident red-shift was seen in the maximal absorption wavelength, transitioning from 337 to 340 nm for MDPs-T. It is noteworthy that proteins frequently feature three aromatic amino acids that encompass tryptophan (Try), tyrosine (Tyr), and phenylalanine (Phe), playing notable functions in fluorescence emissions. These intricate alterations in fluorescence characteristics manifest under the illuminating influence of ultraviolet lamps. The intrinsic fluorescence results showed that the maximum emission wavelengths of both of MDPs-ND and defatted MDPs-ND were 340 nm. The maximum emission wavelengths of MDPs-T and defatted MDPs-T were 338 and 323 nm, respectively.

#### 2.3.3. FT-IR Spectrometry

FT-IR stands as an indispensable tool for unraveling the structural intricacies of active ingredients and their polymers. The efficacy of FT-IR in delineating the secondary structure and chemical groups of proteins was contingent upon the distinctive bands, notably the amide I (1700 to 1600 cm^−1^), amide II (1550 to 1530 cm^−1^), and amide III (1300 to 1260 cm^−1^) bands. Amide I, in particular, serves as a sensitive indicator of protein secondary structures, encompassing α-helix, β-turn, β-sheet, and random coil conformations. The FT-IR spectra analyses of the MDPs-T and MDPs-ND results are elucidated in Figure 4c, alongside detailed data in Table 4.

Broadly speaking, the infrared spectra profiles of the four protein products exhibit notable parallels. Distinctive peaks characteristic of proteins were observed in the MDPs, including peaks at 3297, 2963, 2866, 1745, 1666, 1404, 1250, and 1049 cm^−1^. The signature peaks within the range of 3000 to 3750 cm^−1^ are attributable to the stretching vibrations of N–H and O–H, indicative of hydrogen bonding within the peptide backbone. Therefore, these hydrogen bonds serve as indispensable elements in preserving the structural stability of protein secondary conformations.

Moreover, the absorption peaks spanning the 2800 to 3000 cm^−1^ range, corresponding to the symmetric and asymmetric stretching vibrations of C–H bonds, delineate the hydrophobic region of the protein. Interestingly, both MDPs-ND and MDPs-T present a distinctive peak at 2963 cm^−1^, aligning with the asymmetric extension of CH_2_ primarily found in the aliphatic side chain of proteins. This intricate analysis of the FT-IR spectra unveils profound insights into the structural attributes and chemical properties of MDPs, thereby enriching our understanding of their molecular behavior and potential applications in diverse fields.

The amide I band serves as a critical indicator for assessing protein secondary structure, particularly highlighting α-helix structures (1650 to 1658 cm^−1^) and random coils (1640 to 1650 cm^−1^) [28,29]. At 1666 cm^−1^, an absorption peak indicative of the stretching vibration of the C=O bond could be identified, while the peak at 1404 cm^−1^ could be attributable to both the bending vibration of the N–H bond and the stretching vibration of the C–N bond. The outcomes unveiled the presence of five distinct peaks, pinpointed at the following specific wavenumbers: 3297, 2866 to 2963, 1745, 1666, and 1250 cm^−1^, delineating the regions of amides A, B, I, II, and III, respectively. These findings resonate with the unique stretching and bending vibrations intrinsic to the protein backbone, findings that are extensively corroborated by numerous researchers in the field [15,17].

Regarding the secondary structure analysis of MDPs from the infrared spectrum, additional processing steps are essential, including baseline correction, second-order derivative fitting, and Gaussian deconvolution. This precise approach allows for the quantification of each secondary structure based on the calculation of peak areas. Within the 1600–1700 cm^−1^ range, a multitude of bands emerges, each indicative of distinct secondary structures. Specifically, peaks observed at 1635, 1645, 1655, and 1667–1673 cm^−1^ are associated with β-sheet, random coils, α-helix, and β-turns, respectively. For MDPs-ND, the predominant secondary structure identified is β-fold (33.34%), closely followed by β-turn (31.40%), α-helix (18.40%), and random coils (16.86%). Conversely, for defatted MDPs-ND, the dominant structure shifts towards β-turn (40.80%), succeeded by β-fold (30.02%), α-helix (15.25%), and random coils (13.93%). Upon comparison, it becomes evident that the secondary structures of MDPs-T and defatted MDPs-T exhibit striking similarities, underscoring the preservation of structural characteristics despite the defatting process. This detailed analysis sheds light on the intricate interplay of structural components within MDPs, offering valuable insights into their molecular architecture and potential functional properties.

#### 2.3.4. Wide-Angle XRD Experiment

XRD analysis stands as a fundamental method for discerning the crystallinity or amorphous nature of powders, often serving as a vital tool in probing protein conformation. In the context of MDPs, their crystal structure was precisely figured out through XRD analysis. The resulting data, as depicted in Figure 4d, have unveiled distinctive diffraction peaks at 2θ values of 20°, 28°, 41°, and 50°. This notable pattern suggests that both MDPs-T and MDPs-ND share similar crystal structures. Notably, MDPs-ND exhibited a sharp diffraction peak, a characteristic indicative of its high crystallinity and well-organized structure. Conversely, the presence of diffuse broad peaks is a hallmark of an amorphous structure, whereas crystalline polymers typically manifest as a series of sharp peaks. These findings underscore the crystalline nature of MDPs and resonate with the observations reported by Xu et al., further reinforcing the structural integrity and characteristics of MDPs elucidated through the XRD analysis [19].

#### 2.3.5. Molecular Weight Distribution of MDPs

**Figure 5:**
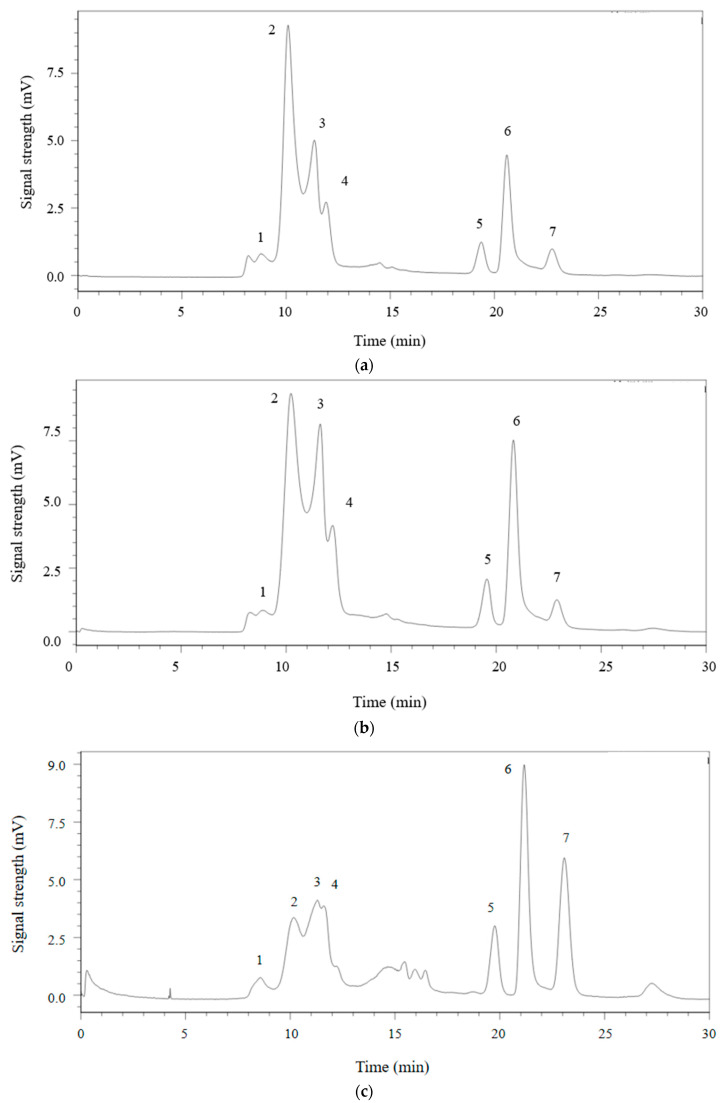

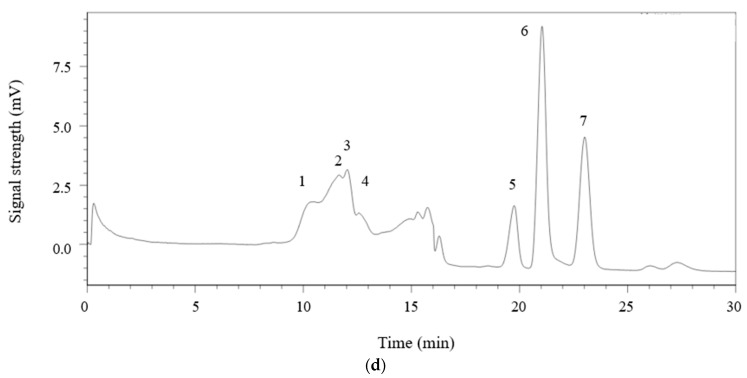
The weight distribution curves of MDPs. (**a**) MDPs-ND; (**b**) Defatted MDPs-ND; (**c**) MDPs-T; (**d**) Defatted MDPs-T. Peak 1: 9.12 × 10^3^ kDa; peak 2: 3.96 × 10^3^ kDa; peak 3: 1.70 × 10^3^ kDa; peak 4: 1.23 × 10^3^ kDa; peak 5: 8.68 kDa; peak 6: 4.26 kDa; peak 7: 1.02 kDa.

As a pivotal physicochemical characteristic, *Mw* can significantly influence the pharmacological activities of proteins. HPGPC was utilized to indicate the *Mw* of MDPs. Utilizing the following equation derived from the standard curve: Log*Mw* = −3.1305 T + 31.548 (R^2^ = 0.999)—where *Mw* represents the weight-average *Mw* and T denotes the retention time—the MDPs were found to consist of molecules with weights of 9.12 × 10^3^, 3.96 × 10^3^, 1.70 × 10^3^, 1.23 × 10^3^, 8.68, 4.26, and 1.02 kDa, corresponding to retention times of 8.79, 10.10, 11.36, 11.92, 19.36, 20.60, and 22.78 min, respectively. The HPGPC spectrum revealed distinct peaks for MDPs, each representing a varying percentage of the total. Relative to the defatted MDPs-ND, there was a slight decrease in the percentage of peak 1, peak 2, and peak 7 for MDPs-ND, while the percentages of peak 3, peak 4, peak 5, and peak 6 exhibited slight increases (Figure 5 and Table 5). Similarly, when contrasted with the defatted MDPs-T, there was a slight decrease in the percentage of peak 1, peak 2, peak 3, and peak 4 for MDPs-T, while the percentages of peak 5, peak 6, and peak 7 showed slight increases.

#### 2.3.6. Amino Acid Analysis of MDPs

The amino acid profile stands out as a paramount factor in determining the quality of natural proteins. As illustrated in Table 6, the contents of 17 amino acids within the MDPs were precisely analyzed. The functional properties of a protein or peptide largely hinge on their amino acid composition. Notably, no significant differences were observed in the amino acid compositions between MDPs-T and MDPs-ND. Both variants boasted a high nutritional value, making them exceptional protein sources. The amino acid profile revealed that glutamic acid was the most abundant amino acid across both MDPs-T and MDPs-ND. However, there was a notable difference in the percentage of essential amino acids between the two. MDPs-T and defatted MDPs-T exhibited percentages of 44.81% and 41.42%, respectively, surpassing the FAO/WHO recommended model of 40%. In contrast, MDPs-ND and defatted MDPs-ND demonstrated percentages of 38.48% and 37.96%, respectively. Furthermore, it is noteworthy that the total amino acid content of MDPs-T and defatted MDPs-T exceeded that of MDPs-ND and defatted MDPs-ND. This comprehensive amino acid analysis underscores the nutritional richness of MDPs-T and defatted MDPs-T, positioning them as highly valuable protein sources.

#### 2.3.7. SEM Analysis of MDPs

**Figure 6:**
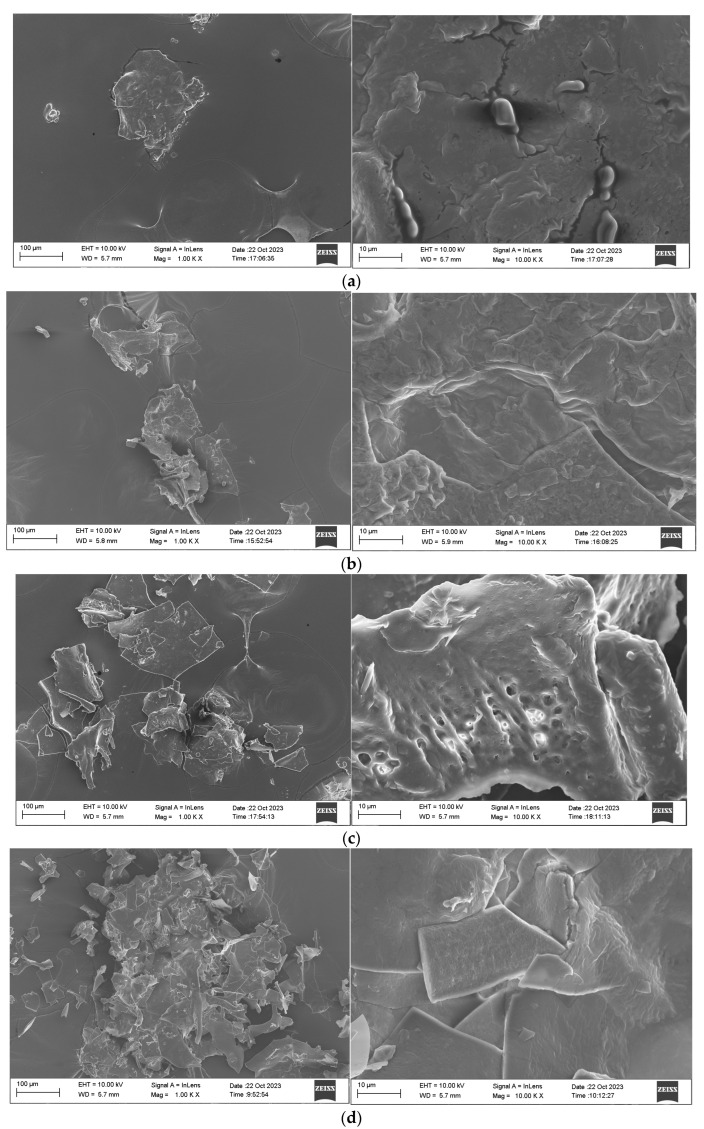
SEM analysis of MDPs: (**a**) MDPs-ND; (**b**) Defatted MDPs-ND; (**c**) MDPs-T; (**d**) Defatted MDPs-T.

SEM emerged as a pivotal tool for the detailed analysis of protein surface structures and morphologies, offering a direct pathway to figure out polymer architectures. The microphology of MDPs-T, defatted MDPs-T, MDPs-ND, and defatted MDPs-ND was precisely examined through SEM, as depicted in Figure 6. Evident differences in both the dimensions and configurations of these materials’ surfaces were identified. At a magnification of 1000-fold, the particles of MDPs-T and defatted MDPs-T presented a striking resemblance to glass shards. Upon closer inspection, the surfaces of MDPs-T exhibited a rough texture with a consistent thickness. Conversely, MDPs-ND and defatted MDPs-ND were shaped like slices under the same magnification. Further magnification to 10,000-fold revealed the rough surfaces of MDPs-ND, characterized by nonuniform thicknesses. Notably, all of the MDPs were found to harbor irregularly shaped, sizable particles exceeding 10 μm in diameter. This comprehensive SEM analysis unveils a nuanced view of the diverse surface structures and morphologies inherent in these materials [30]. Further research is needed for the biological activities and edibility of MDPs-T.

## 3. Materials and Methods

### 3.1. Materials

The *M. domestica* larvae utilized in this investigation were attained from ChanBao Biotechnology Co., Ltd. (Hegang, China; October 2022). All additional reagents employed in this study were sourced as analytical purity or higher and were attained from local suppliers.

### 3.2. Preparing MDPs

The *M. domestica* larvae were specifically fed a diet consisting of healthy toad corpses, resulting in the isolation of proteins referred to as MDPs-T. In contrast, for comparative analysis, commercially farmed larvae were obtained and were fed on a diet comprising vegetables and mixed grains, with the resultant proteins named MDPs-ND. Subsequently, the larvae underwent standard processing procedures. To elaborate further, the larvae were initially cleansed with distilled water, followed by freeze-drying at −30 °C. They were precisely ground into fine particles using a crusher (FW177) attained from Tianjin (China), and the resulting powder was sifted through a 40-mesh sieve to obtain M. domestica larvae powder. This powder was thereafter subjected to defatting with petroleum ether to yield defatted MDPs-T and MDPs-ND. Simultaneously, another portion of the powder was directly dried without defatting to produce MDPs-T and MDPs-ND in their original state. Subsequently, all four types of powder were subjected to drying in a hot air oven at 50 °C and stored at −18 °C until required for further analysis. The dried *M. domestica* larvae powder was precisely mixed with alkaline water in a beaker, then gently simmered for a duration of 0.5 to 2.5 h at temperatures ranging from 20 to 40 °C. The resulting MDPs were attained through centrifugation, specifically at 1000× *g* with H2050R (Xiangyi Instruments Co., Ltd., Changsha, China) for 15 min at a temperature of 10 °C. These samples were carefully stored in a −80 °C freezer until further utilization [31].

The *M. domestica* larvae powders (40.0 g) were subjected to extraction under the following precisely designed conditions: a material-to-liquid (MTL) ratio (X_1_) ranging from 1:25.0 to 1:45.0 g/mL, pH (X_2_) varying from 8.0 to 12.0, extraction time (X_3_) spanning from 0.5 to 2.5 h, and extraction temperature (X_4_) ranging from 20.0 to 40.0 °C. Subsequent to extraction, the pH of each resultant extract was carefully adjusted to neutral utilizing 0.1 M HCl. Dialysis, utilizing a membrane with a molecular weight cutoff of 3500 Da, was thereafter employed over a period of 48 h to eliminate NaCl and other low-molecular-weight substances. Following dialysis, the extraction mixture was subjected to centrifugation, specifically at 8000 rpm with TGL-16D (Jingda Co., Ltd., Changzhou, China) for a duration of 15 min. Through a 0.45-μm filter membrane, we attempted to filter the resulting supernatant, followed by subjecting it to lyophilization with LGJ-18N (Yaxingyike Co., Ltd., Beijing, China). This comprehensive process yielded the crude MDPs, ready for further analysis and characterization.

Quartz cuvettes were precisely utilized for the analysis of the MDPs through a UV–Vis spectrophotometer by U-2901 (Hitachi Co., Ltd., Tokyo, Japan). To ensure accuracy, the instrument underwent zero adjustment with distilled water prior to the measurements being taken. The absorption spectra of the MDPs were subsequently recorded at wavelengths of 260 and 280 nm, following the established method presented by Puray and Villaber. For the quantification of the protein concentration in the MDPs and the determination of MDPs yield, the following equations were employed [32]:

Protein concentration (mg/mL) = (1.55 × A_280_) − (0.76 × A_260_) (1)

Yield of MDPs (%) = (C × V)/m(2)

where A_280_ is the absorption of MDPs at 280 nm, A_260_ is the absorption of MDPs at 260 nm, C is the protein concentration of the MDPs (mg/mL), V is the volume of the MDPs’ extract (mL), and m is the amount of *M. domestica* larvae powder (mg).

### 3.3. Experimental Design of RSM

The experimental design was constructed on the basis of the BBD for the comprehensive RSM study. To figure out the optimal extraction conditions for MDPs, response surface designs with 3 levels (−1, 0, and +1) were strategically implemented. This involved a thorough exploration of various extraction parameters utilizing a BBD comprising 4 factors and 3 levels, resulting in a sophisticated second-order polynomial regression model. Upon analyzing the outcomes of the single-factor tests (Figure 1), the independent variables, involving the MTL ratio (X_1_), pH (X_2_), extraction time (X_3_), and temperature (X_4_), were precisely identified. Subsequently, the proper ranges for these variables were examined and established as appropriate levels for the subsequent experiments. The comprehensive design consisted of 29 intricate combinations, with a strategic inclusion of 5 replicates particularly at the central point, as illuminated in Table 1. To ensure the robustness and accuracy of the outcomes, we attempted to examine all combinations in triplicate.

### 3.4. Characterization of MDPs

#### 3.4.1. UV Spectrum and Intrinsic Fluorescence Analysis

The process commenced with dissolving 10.0 mg of the dried MDPs samples in 5.0 mL of deionized water, followed by a comprehensive scan to identify their maximum absorption wavelength across the extensive range of 200 to 800 nm. This crucial step was conducted with a U-2901 UV–Vis spectrophotometer attained from Hitachi Co., Ltd., with the resulting UV spectra being precisely recorded and analyzed via the professional Origin Pro 9.0 Software.

In pursuit of a deeper comprehension of the intrinsic fluorescence properties of MDPs, we attempted to prepare a solution with a concentration of 1.0 mg/mL in distilled water at a neutral pH of 7.0. This precisely prepared solution was thereafter subjected to a comprehensive analysis via an F-7100 FL spectrophotometer, attained from Hitachi Co., Ltd. The experimental setup involved a precisely controlled scanning speed of 5 nm/s, setting the excitation wavelength particularly at 280 nm. For the emission spectra, a range from 300 to 450 nm was scanned with the same scanning speed. To maintain accuracy throughout the measurements, for both the excitation and emission processes, a uniform slit width of 5 nm was consistently exerted.

#### 3.4.2. Particle Size Distribution and Zeta Potential

The process commenced with the MDPs solutions being precisely diluted to a concentration of 1.0 mg/mL through distilled water. Thereafter, each protein sample was dispensed into dedicated cuvettes. The comprehensive analysis of particle size distribution and zeta potential was undertaken with a laser light scattering in situ particle size analyzer (NanoBrook 90Plus, Brookhaven Co., Ltd., New York, NY, USA) and maintained at a controlled temperature of 25 °C. To ensure precision and reliability, we attempted to thrice repeat the tests for each sample.

#### 3.4.3. FT-IR Spectra

The FT-IR spectra of the MDPs were attained utilizing an advanced FT-IR spectrophotometer (NEXUS-470, Nicolet Co., Ltd., Madison, WI, USA). To prepare the samples, we attempted to mix 10.0 mg of the dried MDPs with 100.0 mg of KBr, and this mixture was finely ground into a powder of homogenous consistency. Subsequently, the powder mixtures were precisely pressed into pellets with a precise diameter of 1 mm. For the FT-IR spectral analysis, the scanning of the prepared pellets was undertaken across the extensive frequency range of 500 to 4000 cm^−1^. This scanning process was accurately repeated a total of 10 times to ensure the accuracy and reliability of the obtained spectra.

#### 3.4.4. XRD Analysis

The XRD patterns of the MDPs were acquired through a methodology presented by Li et al., accompanied by subtle modifications [33]. This analytical process confirmed the crystal structure of the MDPs and was executed utilizing an advanced XRD instrument (D8 advance, Bruker, Luken, Germany). The instrument was configured with a Cu Kα radiation source, set to operate at 40 kV and 40 mA, which may assist in precise quantification. Regarding data collection, the XRD instrument implemented scanning through an angular range from 5° to 80° at a controlled scanning speed of 5° per minute. This strategy ensured a thorough and detailed analysis of the crystalline structure of the MDPs.

#### 3.4.5. Molecular Weight Distribution of MDPs

The MDPs solutions were prepared at a concentration of 2.0 mg/mL by their precise filtration through a 0.22-μm membrane (Shenghan Co., Ltd., Qingdao, China) to ensure the removal of any impurities. Subsequently, these filtered MDPs solutions were introduced into a sophisticated HPGPC system (LC-20AD, SHIMADZU, Kyoto, Japan) for detailed molecular analysis. The HPGPC system utilized in this study featured a G-3000 PWXL ultra-hydrogel linear gel filtration column (Tosoh Co., Ltd., Tokyo, Japan) in combination with an ultraviolet detector to facilitate precise detection and quantification. The elution of the samples was undertaken with deionized water as the eluent. To accurately determine the average molecular weight (*Mw*) of the MDPs, we attempted to draw a calibration curve via a comprehensive set of standards with known molecular weights (13,050, 36,800, 64,650, 135,350, 300,600, and 2,000,000 Da). This precise calibration process enabled a precise and detailed assessment of the *Mw* of the MDPs [34].

#### 3.4.6. Analysis of Amino Acid Composition

The comprehensive investigation into the amino acid composition of MDPs was undertaken on the basis of the methodology outlined by Tian, accompanied by modifications [35]. We attempted to analyze the amino acids with an 835-50 automatic acid analyzer, attained from Hitachi. For this analysis, the total amino acid composition of the MDPs was precisely evaluated following a hydrolysis process, particularly at 110 °C for 24 h, utilizing 6 M HCl. The utilization of external standards played a notable function in the precise quantification of amino acids.

#### 3.4.7. SEM

We attempted to analyze the intricate microstructure of the MDPs through an EVO18 scanning electron microscope (Zeiss, Oberkohen, Oberkochen, Germany) at both 200× and 1000× magnifications. To ensure optimal conductivity for imaging, the MDPs powder was precisely affixed to the sample stage utilizing a conductive adhesive. Thereafter, a precise sputter coating with gold powder was applied for a duration of 1 min before observation.

### 3.5. Statistical Analysis

The expression of data was in the form of mean ± standard deviation, which were attained from a minimum of 3 independent experiments. The statistical analysis was conducted with SPSS 13.0 software developed by IBM (International Business Machines Corporation, Amonk, NY, USA). The comparable analysis of differences, particularly among diverse groups, was implemented through a one-way analysis of variance (ANOVA), supplemented with either an independent sample *t*-test or Fischer’s F-test. A *p* threshold below 0.05 was suggestive of statistical significance.

## 4. Conclusions

The current investigation concentrated on optimizing MDPs-T extraction, achieving a substantial yield of 18.3% under ideal parameters (MTL ratio of 1:36.0 g/mL, pH of 10.0, temperature of 31.0 °C, and 1.3 h). Subsequent analyses using UV, FT-IR, Gaussian fitting, XRD, HPGPC, and SEM methods unveiled strikingly similar physical and chemical properties between MDPs-T and MDPs-ND. However, the following discernible disparity emerged: MDPs-T exhibited a higher proportion of low-molecular-weight components relative to MDPs-ND. A further assay unveiled that both MDPs-T and defatted MDPs-T exhibited an enriched content of α-helix structures, coupled with a diminished presence of β-folds. Moreover, the comprehensive amino acid profiling confirmed the abundant presence of essential and total amino acids in MDPs-T. These notable outcomes underscore the potential of MDPs-T as a valuable protein reservoir. This not only shed light on the remarkable correlation of *M. domestica* in toad feeding, but also provided a robust reference for its holistic utilization.

## Figures and Tables

**Table 1:** Four-factor central composite design matrix and the response values for the extraction rate (%).

Run	Ratio of Material to Liquid (g/mL) X_1_	pH X_2_	Extraction Time (h) X_3_	Extraction Temperature (°C) X_4_	MDPs Yield (%)
1	1:35	9	1.5	25	14.6
2	1:30	9	1.5	30	14.3
3	1:35	10	1.5	30	18.5
4	1:35	10	1.5	30	17.9
5	1:35	11	1.5	25	16.6
6	1:35	10	2.0	35	15.5
7	1:40	10	1.5	35	16.3
8	1:40	9	1.5	30	15.4
9	1:40	11	1.5	30	17.3
10	1:40	10	1.5	25	15.0
11	1:35	10	1.0	25	15.2
12	1:30	10	2.0	30	15.3
13	1:40	10	2.0	30	16.1
14	1:35	9	1.0	30	15.6
15	1:40	10	1.0	30	17.0
16	1:35	10	2.0	25	16.6
17	1:30	11	1.5	30	16.3
18	1:35	10	1.0	35	16.9
19	1:35	9	2.0	30	15.5
20	1:30	10	1.5	35	15.9
21	1:35	10	1.5	30	18.2
22	1:35	10	1.5	30	18.3
23	1:35	9	1.5	35	16.4
24	1:35	10	1.5	30	18.1
25	1:30	10	1.5	25	14.2
26	1:35	11	1.0	30	17.2
27	1:30	10	1.0	30	15.7
28	1:35	11	1.5	35	15.9
29	1:35	11	2.0	30	17.1

**Table 2:** Analysis of variance (ANOVA) of the regression parameters.

Parameter	Sum of Square	df	Mean Square	F-Value	*p*-Value	Significance
Model	45.06	14	3.22	14.93	<0.0001	**
X_1_	2.44	1	2.44	11.31	0.0046	**
X_2_	6.06	1	6.06	28.12	0.0001	**
X_3_	0.129	1	0.129	6.00	0.0280	*
X_4_	1.72	1	1.72	8.00	0.0134	*
X_1_X_2_	0.0021	1	0.0021	0.0096	0.9233	-
X_1_X_3_	0.0415	1	0.0415	0.1926	0.6675	-
X_1_X_4_	0.0309	1	0.0309	0.1433	0.7107	-
X_2_X_3_	0.0034	1	0.0034	0.0158	0.9019	-
X_2_X_4_	1.43	1	1.43	6.62	0.0221	*
X_3_X_4_	1.93	1	1.93	8.97	0.0097	**
X_1_^2^	16.64	1	16.64	77.20	<0.0001	**
X_2_^2^	8.71	1	8.71	40.42	<0.0001	**
X_3_^2^	3.78	1	3.78	17.56	0.0009	**
X_4_^2^	15.33	1	15.33	71.73	<0.0001	**
Residual	3.02	14	0.2155			
Lack of fit	2.04	10	0.2040	0.8341	0.6304	-
Pure error	0.9781	4	0.2445			CV% = 2.82
R-Squared	0.9372		Adj.R-squared	0.8745		

* Significant at 0.01 < *p* < 0.05, ** significant at *p* < 0.01; Source: ANOVA using Design-Expert 7.0.0.

**Table 3:** The particle size distribution and zeta potential of MDPs.

Samples	Particle Size Distribution	Zeta Potential
MDPs-ND	363.2 ± 16.6	−37.0 ± 1.7
Defatted MDPs-ND	135.7 ± 3.3	−46.0 ± 3.4
MDPs-T	246.4 ± 5.6 **^##^	−39.0 ^##^ ± 1.5
Defatted MDPs-T	219.3 ± 31.1	−8.3 ± 1.1

Compared with MDPs-ND, ** significant at *p* < 0.01; Compared with defatted MDPs-T, ^##^ significant at *p* < 0.01.

**Table 4:** The secondary structure fitting results of MDPs.

	Content of Each Component in the Amide I Band/%			
	α-Helix	β-Fold	β-Turn	Random Coil
MDPs-ND	18.40	33.34	31.40	16.86
Defatted MDPs-ND	15.25	30.02	40.80	13.93
MDPs-T	43.55	28.67	27.78	-
Defatted MDPs-T	43.05	28.34	28.60	-

**Table 5:** The molecular weight distribution of MDPs.

Samples	MDPs-ND	Defatted MDPs-ND	MDPs-T	Defatted MDPs-T
Peak 1 (9.12 × 10^3^ kDa)	3.28%	2.24%	6.24%	-
Peak 2 (3.96 × 10^3^ kDa)	38.68%	28.78%	10.30%	6.31%
Peak 3 (1.70 × 10^3^ kDa)	20.22%	24.41%	12.34%	10.37%
Peak 4 (1.23 × 10^3^ kDa)	10.31%	11.54%	11.44%	11.14%
Peak 5 (8.68 kDa)	4.92%	6.04%	9.46%	10.31%
Peak 6 (4.26 kDa)	18.62%	23.42%	31.65%	40.05%
Peak 7 (1.02 kDa)	3.96%	3.57%	18.57%	21.83%

Note: - represents not detected.

**Table 6:** The results of amino acid composition analysis of MDPs.

Amino Acid	MDPs-ND (mg/mL)	Defatted MDPs-ND (mg/mL)	MDPs-T (mg/mL)	Defatted MDPs-T (mg/mL)
Aspartic acid	0.595	0.389	1.055	0.745
Glutamic acid	1.609	1.098	1.322	1.197
Serine	0.283	0.191	0.373	0.316
Glycine	0.331	0.197	0.337	0.318
Histidine	0.801	0.486	0.708	0.720
Arginine	0.387	0.240	0.452	0.413
Threonine	0.228	0.157	0.369	0.301
Alanine	0.337	0.221	0.410	0.491
Proline	0.318	0.192	0.320	0.294
Tyrptophan	0.491	0.314	0.993	0.725
Valine	0.323	0.209	0.510	0.398
Methionine	0.014	0.024	0.276	0.142
Leucine	0.259	0.147	0.600	0.404
Isoleucine	0.166	0.102	0.382	0.280
Phenylalanine	0.561	0.376	0.801	0.514
Lysine	0.441	0.270	0.753	0.524
Cystine	0.023	0.017	0.040	0.037
Trptophan	0.091	0.036	0.117	0.108
Essential amino acids	2.793	1.771	4.399	3.283
Total amino acids	7.258	4.666	9.818	7.927

## Data Availability

Data are contained within the article.
